# Smartphone Applications in the Management of Parkinson's Disease in a Family Setting: An Opinion Article

**DOI:** 10.3389/fneur.2021.668953

**Published:** 2021-05-21

**Authors:** Ting Zhang, Li Dong, Hua Jing, Song Gao

**Affiliations:** ^1^Department of Chinese Medicine, Naval Special Medical Center, Naval Medical University, Shanghai, China; ^2^School of Kinesiology, Shanghai University of Sport, Shanghai, China

**Keywords:** smartphone applications, Parkinson's disease, medicine management, rehabilitation, limitations

## Introduction

With the advent of the twenty-first century, the global population is aging at an unprecedented rate. European countries, such as Portugal, Germany, and Italy, are the hardest-hit areas (1). The aging population profoundly affects the economic growth and pattern of resource allocation accompanied by high-risk neurodegenerative diseases, such as Parkinson's disease (PD) (2, 3).

PD is a common progressive neurodegenerative disease in the elderly. Its main clinical manifestations include resting tremor, muscle stiffness, postural instability, freezing gait, and bradykinesia (4). Moreover, non-motor symptoms may appear at the early phase of PD (5, 6). Studies have shown that the incidence of PD is closely related to age and male patients are more susceptible than female patients (7, 8). By 2030, ~9 million people over the age of 50 will receive a diagnosis of PD in the world, and China will account for half of this population (9, 10). PD not only brings a great physical and psychological burden to the individuals with PD but also greatly reduces the family members' quality of life in long-term family care (2, 11). PD severely reduces cognitive and memory abilities, which often cause difficulties, such as irregular or wrong medication. As is known to all, bradykinesia is the main feature of PD, which may lead to poor compliance with the daily rehabilitation recommended by their doctors (12). Therefore, PD urgently needs a way of medication management and rehabilitation training guidance in a family setting.

However, early diagnosed patients as well as those at the middle stages of PD can still manage this disease specifically by regulating medication and changing their lifestyles with little or no support (13). An active home care plan is an effective strategy to cope with PD, helping individuals with PD manage their condition, improve their quality of life, and live independently (14). The high-speed development of mobile health (mHealth) provides a good prospect for the management of chronic diseases. There are more and more elderly people using smartphones in the world, which have become an important prerequisite for patients to actively participate in mHealth (15). At present, mHealth has been used in the fields of hypertension, diabetes, and cardiovascular diseases (16), and it plays a role in improving health management compliance, symptom management, and social support for individuals with chronic diseases (17).

Users and developers are realizing the potential of applications, and it is estimated that there are more than 40,000 health-related applications on the market (18). However, many applications are not designed to meet the specific needs of PD or their caregivers; hence, only a few studies provide distinctive answers (19). Liao et al. (20) also confirmed this finding. They pointed out that elderly people with PD are willing to adopt new technologies only if they meet their needs and expectations. The main obstacles seem to be lack of technical knowledge and skills, negative attitudes toward the use of this technology, and inaccurate perceptions of people with PD (21, 22). Besides, the lack of general clinical trial evidence supporting smartphone applications and interventions that address the needs of the PD population proved to be prominent (23, 24).

Nevertheless, it is possible to manage PD based on smartphone applications (25, 26). Smartphone applications just make up for the lack of disease management in a home setting. With appropriate training and strategies, technology may enable self-management of PD, including medication management and rehabilitation training in the home environment (27). Mobile applications have the potential to improve the accuracy of assessing the severity of PD, which may be more standardized than the traditional subjective assessment (28). The very positive feature of mobile phones is that they do not have any extra burden for the people who use or wear them (29). Due to their ubiquitous nature, mobile phones can collect a large amount of various data (e.g., physiological or behavioral). These data can then provide decisions about treatment options and monitoring responses (30). Compared with active testing, application-based data collection and analysis may be more objective, which also facilitates the evaluation of patients in remote areas (31).

Users of mobile phone applications can easily download software programs to mobile devices with Internet capabilities. These programs can help doctors monitor and evaluate the symptoms of PD and also support the management of the individuals' daily life, including interacting with doctors, arranging their daily activities or making appointments, rehabilitation training, and managing medication (32). Therefore, the purpose of this article is to discuss the potential of smartphone applications for the management of PD in a family setting.

## Recent Findings on the Use of Smartphone Apps for the Management of PD

Although there are only a few randomized controlled studies on the use of smartphone apps for the management of PD (33), smartphone apps have a promising potential to enhance home care plans for individuals with PD, especially to improve their medication and rehabilitation training compliance (34, 35). The meta-analysis shows that smartphone applications have been used in the daily management of many diseases and have achieved good results in medication compliance (36, 37).

PD severely reduces the memory and cognitive abilities of individuals, which increases the risk of overdose, underdose, and forgetting to take the drug (38). Gao et al. (39) designed a mobile application called Care-PD, which is dedicated to solving the problems of medication management for individuals with PD and such glitches that involve failing to take drugs and or taking the wrong medicine. Among them, the medication management function of the smartphone applications is centered on patients and caregivers, encouraging them to actively record medications, thus setting the name of the medication, medication dosage, medication time, and adverse reaction records. Meanwhile, patients can upload their information to the doctor through the application platform, strengthen their interaction with the doctor, adjust the medication plan in time, and strengthen the patient's self-management. The medication reminder function can increase medication compliance (19, 32). Hu et al. (19) also confirmed this point through their research in which 204 individuals with PD aged 52–87 were questioned about their attitudes toward self-management using smartphone applications, and the results were positive. However, their research also found that elderly people with high education and young people are more likely to accept smartphone applications for self-management. Furthermore, a multicenter randomized controlled trial by Lakshminarayana et al. (32) described the positive effects of a 16-week smartphone application use on short-term self-reported medication compliance and quality of clinical consultation in individuals with PD.

In many countries, health care services are mainly provided in a hospital setting, which may cause transportation and time inconveniences to individuals with PD (40). ParkinsonNet in the Netherlands extends the professional management of PD from the hospital to the community and even to the patient's family (41). This revolutionary management method connects PD patients with health care professionals, encourages patients to take the initiative to manage their health, and has achieved results in reducing falls and fractures (42). In addition, this smartphone-based model can provide better care for individuals with PD and save medical care costs. It has attracted the attention of patients and caregivers in many countries (43). Another practical solution, home-centered community-centered integrated care (iCARE-PD), utilizes the potential of smartphone applications and has a direct impact on clinical practice (44). Besides, iCARE-PD increases the chances of caring for individuals with PD by optimizing their cost, especially in rural areas or low-income countries with potential benefits (45).

Recent findings indicate that wearable devices connected to smartphone apps can monitor patients' motion and non-motor symptoms, which is helpful for doctors to have a more comprehensive understanding of the patient's condition (46). PD_Manager is a mobile health platform designed to cover most of the content related to PD's home management (46). Its mechanism includes the use of a bracelet and an insole sensor that connect to a smartphone to monitor the patient without interference, which helps doctors to have a more comprehensive understanding of the patient's condition. As Motolese et al. (25) point out, most patients are satisfied with the monitoring program provided by smartphones, but its substantial effect needs further verification.

Rehabilitation training is an important part of the daily management of PD. Exercise therapy is beneficial to the motor symptoms and balance ability of individuals with PD (47). However, going to the hospital for rehabilitation may involve the inconvenience of transportation or time. Generally speaking, in the early stages of PD, which is Hoehn and Yahr stages 1–2.5, mobile applications can help individuals with PD to achieve rehabilitation training in a home setting (48). It is worth mentioning that smartphone applications can promote the completion of a rehabilitation plan for individuals with PD in a variety of interesting ways.

Ginis et al. (49) conducted a study on gait training for individuals with PD based on a smartphone application (CuPiD-system). Their results revealed that smartphone-based walking feedback training had similar effects to conventional gait training in terms of balance ability and maintaining the quality of life. This benefit can be attributed to real-time feedback and stimulation of corrective actions. Lopez et al. (50) showed that rhythmic music can improve the gait of PD. An application called Listenmee was used in their research to produce auditory rhythm cues that match the patient's step frequency to improve a PD individual's gait. However, the process of visual and auditory cues requires more attention, communication, and spatial orientation than that of tactile cues (51). Tactile cues may be a more effective means to regulate motor cognitive performance. This is the same as the results of the study by Ivkovic et al. (52), who found that the use of smartphone-based tactile cues can adjust the performance of simple (sitting heel flaps) and complex (straight-line walking) motor tasks in individuals with PD.

## Conclusion

During the outbreak of coronavirus disease 2019 (COVID-19), the application value of Internet medical care was fully demonstrated. The smartphone application is an important part of Internet medical care. They are popular with patients for their convenience and low cost. With the continuous improvement of technology, management applications for PD are gradually developed and tested. The authors of this opinion article point out that the management function based on the smartphone app can strengthen medication management and rehabilitation guidance in a home setting and reduce the physical and psychological burden of caregivers. However, the management and evaluation functions of the current applications are only applicable to a small scale, and there is a lack of evidence-based clinical verification on a larger scale. Individuals' compliance with smartphone applications requires further research and observation.

Many smartphone applications are not designed to meet the specific needs of PD. Companies that develop mobile applications related to PD should focus on meeting the specific needs of individuals with PD and strengthen medication management and rehabilitation guidance in a family setting. Building a smartphone-based PD assessment and management model not only provides a possible platform for improving home care plans and quality of life but also helps to solve the negative impact of an aging society and has high scientific and social value.

## Author Contributions

SG and HJ conceived the manuscript and revised the drafts. TZ and LD wrote the first draft. All authors contributed to the article and approved the submitted version.

## Conflict of Interest

The authors declare that the research was conducted in the absence of any commercial or financial relationships that could be construed as a potential conflict of interest.
